# Global mapping of freshwater nutrient enrichment and periphyton growth potential

**DOI:** 10.1038/s41598-020-60279-w

**Published:** 2020-02-27

**Authors:** R. W. McDowell, A. Noble, P. Pletnyakov, B. E. Haggard, L. M. Mosley

**Affiliations:** 1 AgResearch, Lincoln Science Centre, Private Bag 4749, Christchurch, 8140 New Zealand; 2 Faculty of Agriculture and Life Sciences, P O Box 84, Lincoln University, Lincoln, 7647 Christchurch, New Zealand; 3 Biological and Agricultural Engineering Department, University of Arkansas, Fayetteville, AR 72703 USA; 4 School of Biological Sciences, University of Adelaide, Adelaide, SA 5005 Australia

**Keywords:** Environmental impact, Hydrology, Limnology

## Abstract

Periphyton (viz. algal) growth in many freshwater systems is associated with severe eutrophication that can impair productive and recreational use of water by billions of people. However, there has been limited analysis of periphyton growth at a global level. To predict where nutrient over-enrichment and undesirable periphyton growth occurs, we combined several databases to model and map global dissolved and total nitrogen (N) and phosphorus (P) concentrations, climatic and catchment characteristics for up to 1406 larger rivers that were analysed between 1990 and 2016. We predict that 31% of the global landmass contained catchments may exhibit undesirable levels of periphyton growth. Almost three-quarters (76%) of undesirable periphyton growth was caused by P-enrichment and mapped to catchments dominated by agricultural land in North and South America and Europe containing 1.7B people. In contrast, undesirable periphyton growth due to N-enrichment was mapped to parts of North Africa and parts of the Middle East and India affecting 280 M people. The findings of this global modelling approach can be used by landowners and policy makers to better target investment and actions at finer spatial scales to remediate poor water quality owing to periphyton growth.

## Introduction

Periphyton contains a broad range of algae, cyanobacteria, heterotrophic microbes, and detritus that grows on the beds of streams and rivers. Some species of cyanobacterial algae can be toxic, while the excessive growth and subsequent death and decay of toxic and non-toxic species can deplete oxygen, clog the hyporheic zone and alter pH^1^. These changes can impair the reproductive capacity or even kill fish and bottom-dwelling animals, taint potable water supply and reduce the aesthetic and recreational quality of streams and rivers^2^. These effects, commonly termed eutrophication, put aquatic biodiversity and ecosystem function at risk and globally, cost billions of dollars annually to remediate^3,4^. To target efforts to remediate periphyton growth, information is required on where periphyton grows, how much grows, is the level of growth acceptable and what controls growth. This information is commonly available at a site or catchment-scale, but seldom available at a regional or national scale. To our knowledge, no global analysis exists.

The controlling factors important in periphyton growth include light, temperature, flow rates and nutrient concentrations and bioavailability^5–8^. In most streams and rivers, little can be done about altering light, temperature or flow rates to minimise periphyton growth, therefore most attention focuses the relative concentrations and bioavailability of nitrogen (N) and phosphorus (P), although in some system carbon may also be important^9,10^. The ratio of N to P has been found to limit growth, not only in periphyton but also in microbes and terrestrial fauna and freshwater algae^11,12^. Originally described as a molar ratio of carbon (C), N and P of 106:16:1^13^, in marine phytoplankton, the ratio reduces to N and P in freshwater due to an abundance of C^14^. If considered in terms of mass, a N:P concentration ratio> approx. 7:1, suggests growth will be limited by P, whereas if the ratio is <approx. 7:1, then growth is predicted to be N-limited. Without site-specific experiments to establish N- or P-limitation the Redfield ratio can still be used as an indicator of likely periphyton response.

Stressor-response relationships show that the growth of periphyton is proportional to the concentration of the limiting nutrient^15^. Investigations of the public perception of ‘good’ and ‘bad’ water quality suggest that water quality is deemed to be undesirable when the percentage of periphyton cover was >25–30%, equivalent to a chlorophyll-a concentration of 120–200 mg m^−2^ ^16–20^. These chlorophyll-a concentrations have been used in support of threshold concentrations of N and P derived in the US, Europe, China, South America, Africa and Australasia to prevent periphyton growth^15,21^. We review these studies to determine if there is a consistent threshold in N or P that can be used globally in conjunction with estimates of N and P concentration to predict the likelihood of undesirable periphyton growth in catchments where little or no data exist.

There is some evidence that the chemical form of N and P can influence periphyton growth^22^. Both N and P can exist in dissolved (usually defined as that which passes through a 0.45 µm filter) and particulate forms, and in inorganic and organic forms. Dissolved inorganic and some dissolved organic forms of N and P are assumed to be immediately available for periphyton uptake, whereas nutrient ions within and on particles must be released via desorption or enzymatic processes, which reduces their bioavailability^23^. Furthermore, microbial degradation of organic nutrient forms (often comprising much of the total N [TN] and P [TP]) may release dissolved nutrients. In fast-flowing streams and rivers there is less time for particulate forms of N and P to be accessed by periphyton; hence, many stressor-response relationships are better described by dissolved inorganic N to P ratios than total N:P ratios^7^. Conversely, TN:TP ratios are used to describe stressor-response relationships in slow-flowing rivers and standing water with long water residence times where particles settle and can be accessed by microbes which facilitate nutrient dissolution^24^.

To model the likelihood of water quality impairment by periphyton growth, detailed information is required not only about the state of nutrient concentrations during the growth period, but also about factors involved in the loss of N and P from land to water, such as precipitation, soil type and land use^25,26^. There are many models that can predict the concentration, load and yield of N and P at a catchment scale^27,28^ and a few that can predict the load and yield of N and P at a regional or global scale^29,30^. However, models that can predict N and P concentrations at a global scale remain elusive.

We hypothesized that by combining data relating to the limiting nutrient and its bioavailability and concentration, the likely magnitude of periphyton growth can be estimated, enabling landowners and policy makers to more accurately target investment and actions to remediate poor water quality owing to nutrient concentrations and periphyton growth. Targeting actions to a limiting nutrient and to the right place would improve the cost-benefit of actions compared with a non-targeted approach. This knowledge may also help to inform expectations about the rate at which actions will remediate water quality^31^.

The aim of this study was to produce global models of N and P concentrations using N and P data from thousands of sites sampled worldwide between 1990 and 2016, and then to use these data to predict nutrient enrichment, the limiting nutrient and periphyton growth. Specifically, we started by assuming that other controlling factors such as light, temperature and other macro- and micro-nutrients were not limiting growth consistently throughout the year. We derived summary statistics for dissolved and total N and P concentrations and ratios to determine which nutrient was limiting periphyton proliferation during the growing season. We then produced a global model of median N and P concentrations during the growing season to predict where median nutrient concentrations could exceed thresholds and, hence, where periphyton biomass could accumulate at undesirable levels. Finally, we discuss the application of our findings to the management of periphyton biomass.

## Results

### Nutrient concentrations and models

Data in the filtered catchments used in our model calculations cover 56% of the area in the world’s river basins. Significant (*P* < 0.05) variables and their coefficients for the optimised model predicting median nutrient concentrations of NO_3_-N, DRP, TN and TP during the growing season in tropical and temperate regions, but also including boreal and tundra areas above the Arctic circle, are available in the Supplementary Tables S1–S4. The number of predictive variables ranged from 12 for TN to 18 for NO_3_-N and DRP; the model for TP had 16 variables. Parameters found to be significant amongst all models were those describing biomes for temperate and tropical forests, and grasslands and tundra. Variables such as Olsen P, the percentage of cropland in a catchment, mean precipitation and mean slope were found to be significant in models used to predict DRP and/or TP. The percentage of cropland in a catchment and precipitation were also important predictors of median TN concentrations. However, unlike DRP, soil wetness was important for NO_3_-N and TN. The coefficients of determination for predicting NO_3_-N, DRP, TN and TP were 0.49, 0.35, 0.60 and 0.41, respectively.

**Figure 1:**
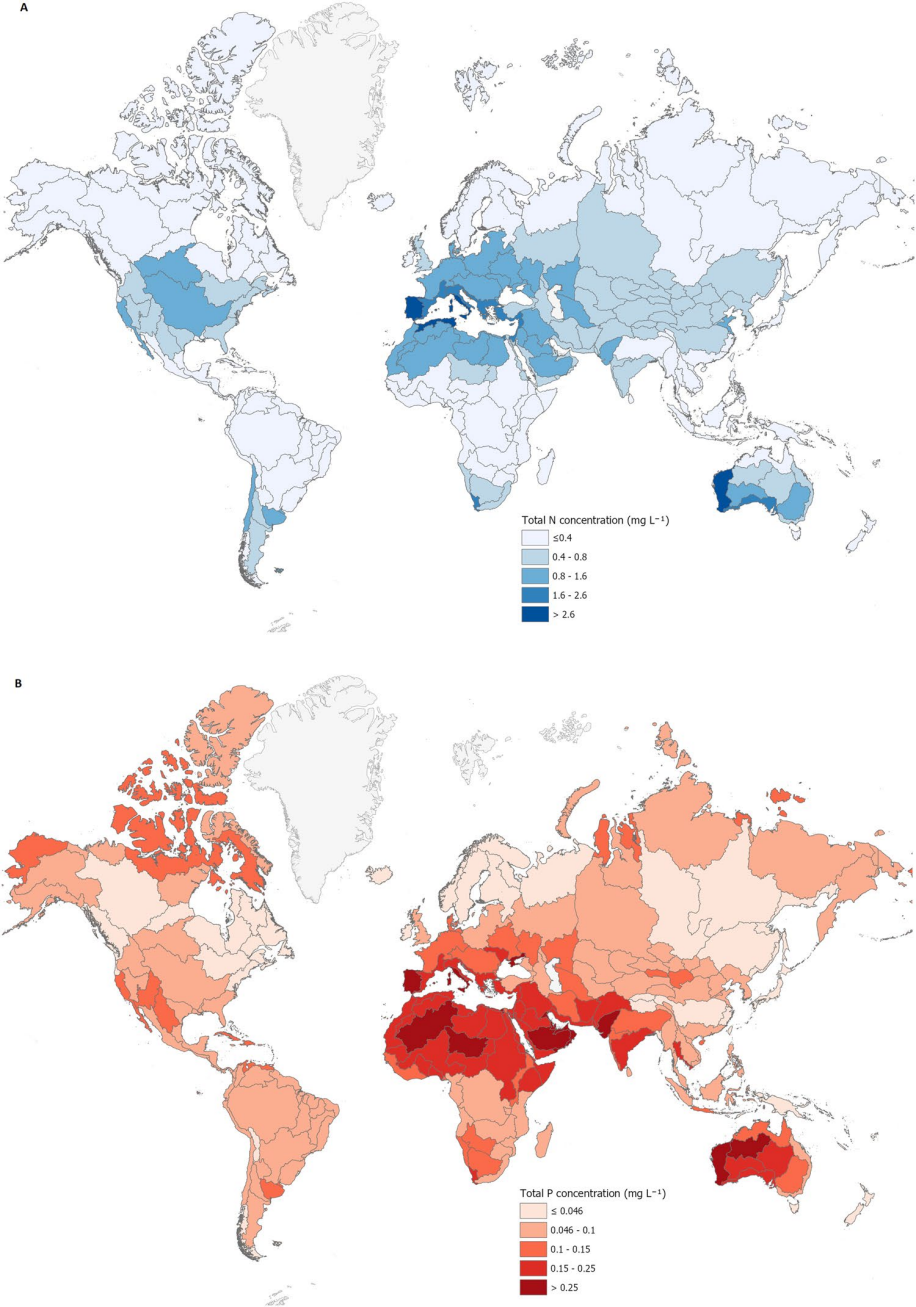
(**A**,**B**) Relative modelled median total P (**A**) and total N (**B**) concentrations (mg L^−1^) during likely periphyton growth periods at catchment scale across the globe. Areas without predictions are white (e.g., Greenland and Antarctica). The relative median values for each of the nutrients are depicted as five different concentration ranges, which include the threshold and half threshold concentration ranges.

Using the predictive models developed, median concentrations for TP, TN, DRP and NO_3_-N during the growing season were projected for catchments across the globe (Fig. 1A,B; Supplementary Fig. S1A,B). Nitrogen fractions tended to be most enriched in temperate regions, particularly in Europe, North America and some areas of Australasia and South America, as well as in some tropical and sub-tropical areas of South and South-east Asia (Fig. 1B and Supplementary Fig. S1B). A somewhat similar pattern was evident for DRP (Supplementary Fig. 1A). However, DRP and particularly TP showed enrichment in dry areas of Africa and the Middle East, and small areas of North America and Asia above the Arctic circle were also enriched (Supplementary Figs. S1A and 1A).

These models are only likely to be valid for large streams and rivers, given that the mean Strahler stream order across our global database was 6.6. Therefore, we next validated our models against catchments of Strahler stream order ≥ 6 in the Murray-Darling and NZWQ databases. In the NZWQ databases, approximately 80% of the catchments were removed, because they represented stream orders of <6. A plot of the observed versus predicted TN and TP concentrations (Supplementary Fig. S2) showed that the coefficients of determination for TN and TP predictions (0.58 and 0.59, respectively) were similar if not better than those across the rest of the globe (0.41 and 0.60, respectively; Supplementary Tables S2 and S4). Furthermore, the slope of the regression line suggested that average TP was approximately 20% under predicted and TN approximately 8% over predicted. We conclude that the nutrient concentration models were therefore robust enough to estimate periphyton growth at a global scale.

### Likely nutrient limitation and likely periphyton growth

**Figure 2:**
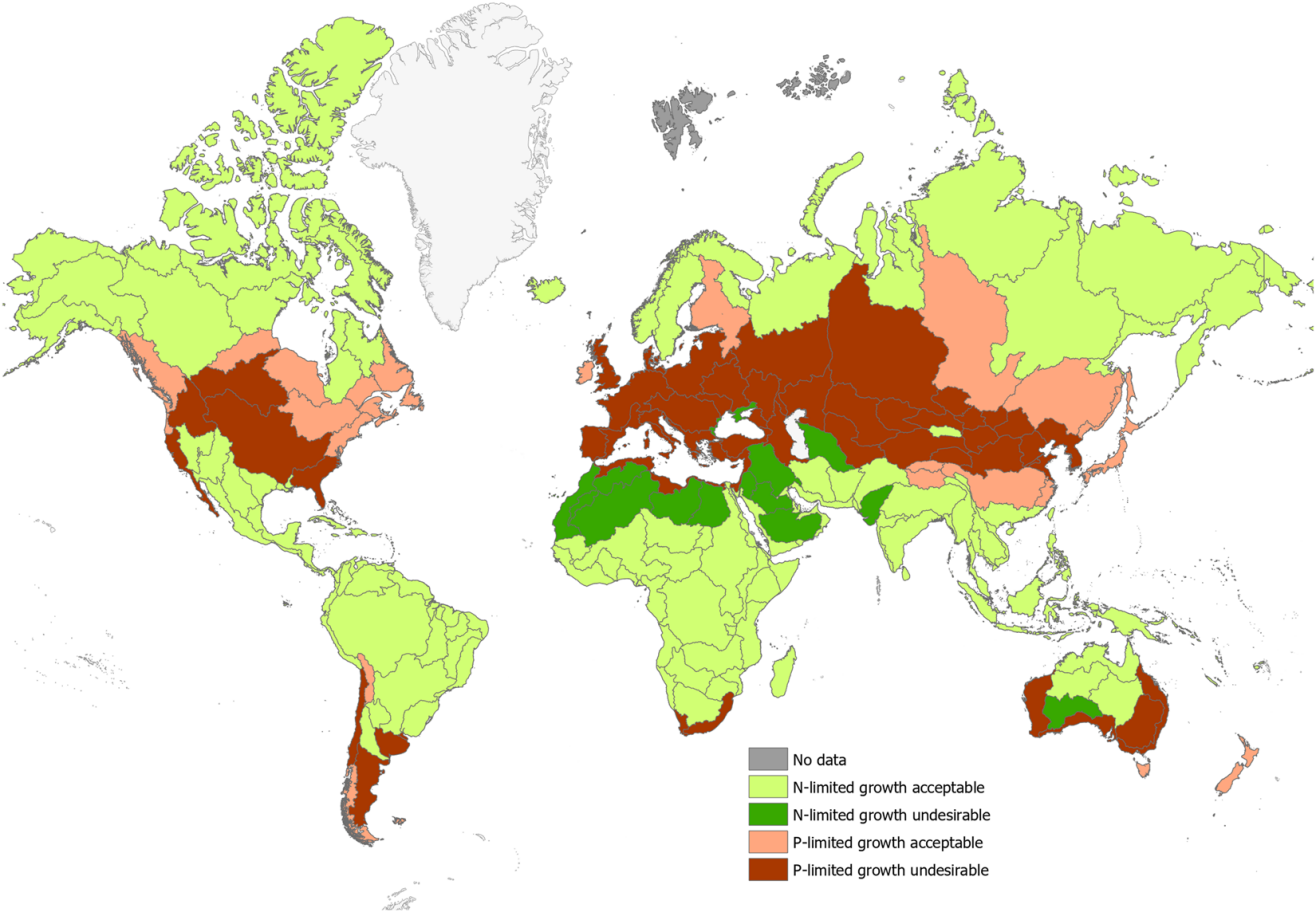
Map showing the large basins predicted to have acceptable periphyton growth and N-limitation (catchment type 1), undesirable periphyton growth with N-limitation (catchment type 2), acceptable periphyton growth and P-limitation (catchment type 3) and undesirable periphyton growth with P-limitation (catchment type 4).

Predicted median nutrient concentrations were used in combination with the global mean TN and TP thresholds to categorize catchments across the globe as either ‘acceptable periphyton growth and N-limitation’ (catchment type 1), ‘undesirable periphyton growth and N-limitation’ (catchment type 2), ‘acceptable periphyton growth and P-limitation’ (catchment type 3) or ‘undesirable periphyton growth and P-limitation’ (catchment type 4) to estimate the prevalence of these four catchment types at a global level. The geographical distribution and estimated area occupied by each of these four catchment types are shown in Fig. 2 and in Table 1 (by continent). We predict that 31% of the global landmass contained catchments that may exhibit undesirable levels of periphyton growth (74% of periphyton growth in these catchments was caused by P-enrichment and 26% by N-enrichment). Most P-limitation and enrichment likely to induce undesirable periphyton growth is predicted to occur in temperate regions, whereas enrichment of N causing undesirable periphyton growth is restricted to North Africa, the Middle East and some parts of South Asia and Australia.

**Table 1:** Estimated area (millions of km^2^) of each continent (percentage in parentheses) predicted to have acceptable periphyton growth and N-limitation (catchment type 1), undesirable periphyton growth with N-limitation (catchment type 2), acceptable periphyton growth and P-limitation (catchment type 3) and undesirable periphyton growth with P-limitation (catchment type 4).

Continent	Type 1	Type 2	Type 3	Type 4
Africa	22.7 (76)	6.1 (20)	0.0 (0)	1.1 (4)
Asia	19.4 (44)	4.2 (9)	8.1 (18)	12.5 (28)
Europe	2.2 (23)	0.0 (0)	0.6 (7)	6.6 (70)
North America	11.7 (53)	0.0 (0)	4.2 (19)	6.1 (28)
Oceania	3.8 (50)	0.8 (11)	0.3 (5)	2.6 (34)
South America	15.6 (88)	0.0 (0)	0.7 (4)	1.5 (9)
World	75.3 (58)	11.1 (9)	14.0 (11)	30.5 (23)

We estimate that 280 million and 1734 million people live in catchments where undesirable growth of periphyton is likely and N limited (catchment type 2) or P limited catchment type 4), respectively (Table 2). Of the former group, most live in Asia, whereas of the latter, most people live in either Asia, Europe or North America (Table 2). Meanwhile, approximately 4131 million people live in catchments where periphyton growth is acceptable and N-limited (catchment type 1) and 970 million people live in catchments where periphyton growth is acceptable and P-limited (catchment type 3).

**Table 2:** Number of people within each continent living in catchments predicted to have acceptable periphyton growth and N-limitation (catchment type 1), undesirable periphyton growth with N-limitation (catchment type 2), acceptable periphyton growth and P-limitation (catchment type 3) and undesirable periphyton growth with P-limitation (catchment type 4).

Continent	Type 1	Type 2	Type 3	Type 4
Africa	1,025,741,239	44,092,182	—	88,646,459
Asia	2,459,163,941	233,927,831	817,010,315	763,406,287
Europe	18,793,629	2,224,451	19,826,163	654,844,514
North America	252,507,691	—	123,627,603	188,139,679
Oceania	617,330	44,262	4,786,156	19,024,132
South America	3,747,21,630	—	4,761,001	39,165,729
World^1^	4,131,193,677	280,244,464	969,519,504	1,734,202,668

^1^Excludes Antarctica and Greenland.

**Figure 3:**
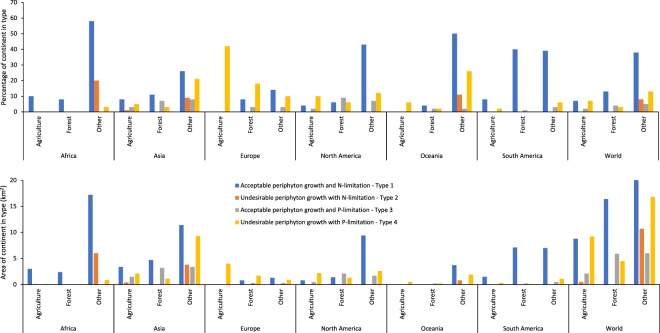
Area (million km^2^) (percentage in parentheses) of either agricultural, forest or other land use within each continent by catchment type. Note that ‘World’ excludes Antarctica and Greenland.

When broken down by land use, for areas where N-limitation and enrichment lead to undesirable periphyton growth, relatively small proportions of the land are used for agriculture or forestry (Fig. 3). In contrast, in areas where P-enrichment causes undesirable periphyton growth, agriculture dominates the land use, particularly in Europe and in North America.

**Figure 4:**
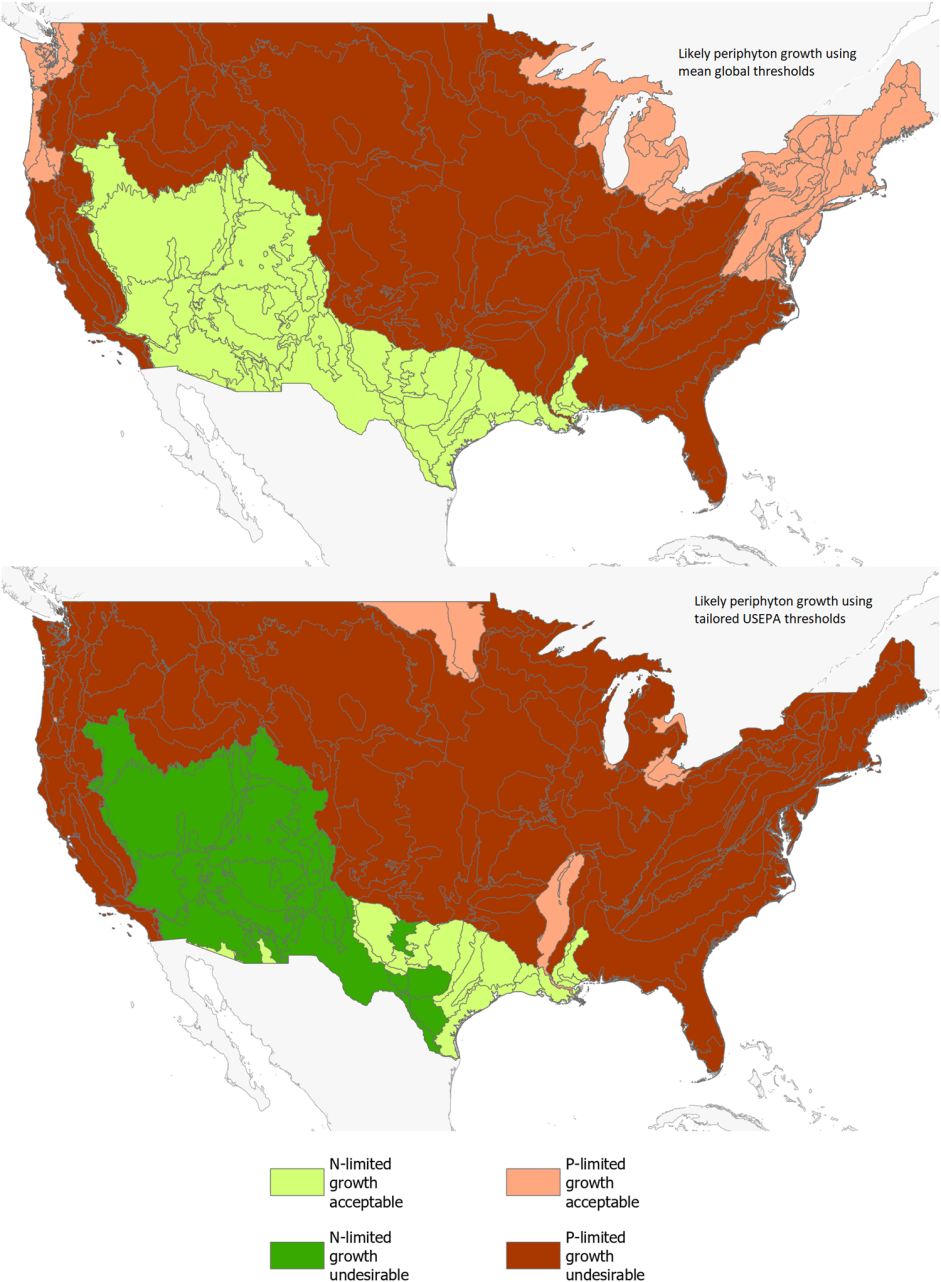
Map showing areas of the US predicted to have acceptable periphyton growth and N-limitation (catchment type 1), undesirable periphyton growth with N-limitation (catchment type 2), acceptable periphyton growth and P-limitation (catchment type 3) and undesirable periphyton growth with P-limitation (catchment type 4) when calculated using global thresholds of 800 and 46 µg L^−1^ for total N and P, respectively (top), and when calculated using USEPA^32^ thresholds for 13 out of 14 ecoregions (bottom).

To validate the geographic representativeness of our global thresholds we compared the estimated areas occupied by the different catchment types predicted using our global thresholds with those predicted when using the thresholds applied to aggregated level III ecoregions in the continental US by the USEPA^32^ (Fig. 4 and Table 3). The USEPA^32^ thresholds are tailored to ecoregions which account for land use and are mapped at a finer scale than biomes^33^. After aligning the global mean thresholds to ecoregions, both the global mean and tailored USEPA thresholds suggest that a large area of the continental US is enriched with nutrients and limited by P. Disparities between the two sets of thresholds were greatest in the southwest and northeast US, accounting for 20% of aerial coverage. Here global mean thresholds tended to under-predict periphyton growth caused by N and P enrichment (Fig. 4 and Table 3). The low level of disparity suggests that our global thresholds can be used at a sub-national scale.

**Table 3:** Total nitrogen and phosphorus thresholds^32^ by aggregated level III ecoregions in the US, and the percent difference between the predicted ecoregion-specific thresholds and the predicted global thresholds (800 and 48 µg L^−1^ of total N and P, respectively) for catchment types 1–4, and the sum area (and % of total area) of cases as calculated by ecoregion and global thresholds.

Aggregate level III ecoregion	Total N threshold (mg L^−1^)	Total P threshold (mg L^−1^)	Ecoregion threshold versus global threshold (% difference)			
			Type 1	Type 2	Type 3	Type 4
I (Willamette and Central Valleys)	0.310	0.047	0	0	0	0
II (Western Forested Mountains)	0.120	0.010	−27	27	−9	9
III (Xeric West)	0.380	0.022	−75	75	0	0
IV (Great Plains Grass and Shrublands)	0.560	0.023	−19	19	0	0
V (South Central Cultivated Great Plains)	0.880	0.067	0	0	−5	5
VI (Corn Belt and Northern Great Plains)	2.180	0.076	0	0	0	0
VII (Mostly Glaciated Dairy Region)	0.540	0.033	0	0	17	−17
VIII (Nutrient Poor Largely Glaciated Upper Midwest and Northeast)	0.380	0.010	0	0	−51	51
IX (Southeastern Temperate Forested Plains and Hills)	0.690	0.037	0	0	−73	73
X (Texas-Louisiana Coastal and Mississippi Alluvial Plains)	0.760	0.128	0	0	48	−48
XI (Central and Eastern Forested uplands)	0.310	0.010	0	0	−15	15
XII (Southern Coastal Plain)	0.900	0.040	0	0	0	0
XIII (Southern Florida Coastal Plain)	—	—	0	0	0	0
XIV (Eastern Coastal Plain)	0.710	0.004	0	0	−52	52
Sum area (and %) of catchment types by ecoregion threshold^1^			484649 (6)	1595808 (20)	239887 (3)	5465312 (70)
Sum area (and %) of catchment types by global threshold^1^			2080457 (27)	0 (0)	831720 (11)	4873479 (63)

^1^Note that sums of percentages do not add up to 100% due to differences in rounding up.

## Discussion

### Hotspots of N and P enrichment

The delivery of nutrients to streams is controlled by a range of climatic, soil and management factors that vary greatly over space and time. We successfully developed models to predict at a global scale how these factors influence nutrient concentrations in freshwaters. Our DRP and TP predictive models incorporate many variables known to influence the delivery of P to waterways, such as increasing concentrations over time of P in soils (e.g., Olsen P)^34^, the percentage of cropland in a catchment^35^, annual precipitation^36^, and mean slope^37^. The percentage of cropland, mean slope and annual precipitation were also incorporated into our model for TN, because these variables were found to significantly influence N concentrations in waterways, which may reflect the involvement of soil erosion processes as they change with elevation^38^. However, both TN and NO_3_-N were positively associated with soil wetness, which may reflect a greater likelihood for N to be lost via leaching and denitrification, as opposed to P, which is more likely to be lost via surface runoff^39^.

All models included biomes as predictors. Recent work has argued that freshwater ecosystems are better described by arrays of multiple biophysical gradients such as precipitation, latitude and longitude than areas with specific boundaries like biomes^40^. However, we contest that while biomes are approximations of edaphic characteristics that span large areas, when combined by nutrient-specific variables these combined models produce gradients compatible to those of Dodds, *et al*.^40^.

Interestingly, both DRP and NO_3_-N models incorporated population density as a predictive variable. The inclusion of this variable could reflect the contribution of point sources, the signature for which tends to be greater under low flow conditions when contributions from diffuse surface runoff and leaching are lower^41^. Such hydrological drought conditions are increasing world-wide due to anthropogenic climate change, water extraction and regulation^42^. A recent analysis has also shown that together with an increase in the amount of land used for agriculture, population growth during the 20^th^ century resulted in an increase in the proportion of dissolved inorganic nutrients relative to total nutrients^43^. Furthermore, based on the current population trajectory, especially in the developing world^44^, undesirable periphyton growth is likely to increase in the future.

Although median concentrations of NO_3_-N and TN paralleled geographical factors such as land use and catchment population density (Fig. 1B, Supplementary Tables 1–4), many of the same factors predicted an enrichment of DRP or TP in some unexpected areas. These included many arid and polar areas that are unlikely to support agriculture (Fig. 1A). Enrichment in dry areas is most likely caused by evaporation, low dilution rates and high productivity, but may also reflect soil water repellency. This generation of soil water repellency can lead to P-laden sediment reaching rivers from infiltration-excess surface runoff, for example following short and sharp rainfall events^45^. In contrast, because of a longer time before runoff, saturation-excess surface runoff tends to carry with it less sediment and therefore less P^36^. The enrichment of DRP and TP in polar regions could be because of increased soil processing (e.g., mineralisation), making P available that was previously locked up by cold conditions, combined with greater transport to rivers caused by snowmelt and runoff^46^.

### Limitations of the analysis

There are several caveats concerning the spatial and temporal representativeness of our analysis. Spatial coverage, although global, is restricted to large catchments (stream order 6 or above) for which data were available. Nevertheless, a recent analysis of 349 headwater streams and 249 large rivers in the UK showed that headwaters were predominantly N-limited, whereas large rivers were P-limited^14^. This shift in limitation was attributed not only to a greater input of nutrients downstream (e.g., through point sources), but also to a greater input of N via groundwater, forcing P-limitation in large rivers where input of P via surface runoff is less evident. Our analysis indicated that at a large scale, the UK was likely to be P-limited, but also P-enriched, which is consistent with the large river findings of Jarvie, *et al*.^14^.

A similar analysis to that performed in the UK was performed in New Zealand involving 1100 sites varying from headwaters to large rivers (Strahler stream order 8)^47^. Of these, 76% of sites were P-limited, but P concentrations were low. Our analysis similarly suggests that large catchments in New Zealand are likely to be P-limited, but not enriched enough to induce undesirable levels of periphyton growth. However, periphyton growth is a common occurrence in small rivers and streams in New Zealand^48^. This suggests that the results of our global analysis should not be expected to be applicable at fine scales, but nevertheless could act as a tool to target areas for further investigation should larger scale catchments be deemed at risk of periphyton growth.

By filtering our data for May to October in the Northern Hemisphere and for November to April in the Southern Hemisphere, we also selected for warmer and brighter conditions when the likelihood of periphyton growth was greatest^49^. However, we did not account for periods of low and stable flow that would allow periphyton to accumulate^5^. We therefore advise that the frequency of high, flushing, flows should also be considered when interpreting our estimates of likely periphyton growth. Similarly, any light limitation to periphyton due to water quality (e.g., high turbidity) may reduce or eliminate undesirable levels of growth^7^. High turbidity is particularly prevalent in dry areas and areas with a high proportion of cropland, intensive grazing or other catchment disturbances like dredging^50–52^.

When calculating the global threshold for likely periphyton growth, the majority of studies we drew upon were conducted in the US, even though more research studies have been undertaken in the European Union^53^. We drew upon US studies because they used a comparable set of methods to determine nutrient threshold levels. We, like others, acknowledge that regionally calibrated models will generally outperform uncalibrated models or models applied at larger scales^54^. However, where it was possible to compare a global threshold to thresholds for US ecoregions, global threshold only underpredicted periphyton growth over 20% of the country (Fig. 4). Our model should therefore be used as a first look to identify the risk of growth and followed up by more detailed local studies where the risk is deemed to be high.

As noted in the Introduction, the Redfield ratio can vary depending on the organism and the study area, and our use of a threshold value in our model therefore has potential limitations for assessing periphyton growth risk. In particular, periphyton can contain N-fixing cyanobacteria that can overcome N limitation^55^. This could mean that streams that we have assessed as N-limited but unlikely to have periphyton growth (catchment type 1), could still achieve undesirable periphyton growth via N-fixation. If this is the case, then the estimated size of the areas and populations affected could be greatly expanded (see catchment type 1 statistics in Table 1).

### Implications for policy and management

Our findings suggest that large populations and areas of the world are affected by nutrient enrichment and undesirable levels of periphyton growth (Table 2), which would lead to the degradation of aquatic ecosystems and decrease recreational values in these regions. Our findings also indicate where the growth of periphyton is likely limited by N or P at large scales. This information could be used to guide regional or national-level actions that: (1) prioritise and tailor good management practices to the limiting nutrient, (2) determine by how much the problem of nutrient loss needs to be mitigated before an acceptable level of periphyton growth is achieved, and (3) determine the likelihood of issues due to nutrient enrichment and undesirable periphyton growth in estuarine or coastal marine areas. Before undertaking such actions, in accordance with best practice, the response of periphyton to nutrient enrichment should be assessed in each target area to reduce uncertainty. However, where resources or practicalities do not allow such assessments (e.g., remote or undeveloped regions), our analysis may serve as a useful default.

In isolating the limiting nutrient (and form thereof) and prioritising areas that require remediation, the choice and cost-effectiveness of good management practices can be optimised^56^. Once land managers know which nutrient to concentrate on they can identify small areas of the farm, often term critical source areas, that account for the majority of losses to water^57^. Concentrating on the limiting nutrient and their areas of loss may remediate periphyton growth faster than an untargeted approach. This is a concept being considered in some jurisdictions; for example, under the Water Framework Directive in the EU^58^ and the National Policy Statement for Freshwater Management in New Zealand^59^. However, while our findings may help to identify the limiting nutrient, it would be wise to mitigate the loss of both N and P to avoid situations where the limiting nutrient changes downstream due to catchment conditions or the sudden loss of the limiting nutrient; for example, via an accidental discharge of nutrient-rich effluent^9^.

Our results might be further investigated to determine whether there is a gap between the targets set for nutrient concentration levels (relating to periphyton growth) and what is observed or estimated to occur (e.g., Fig. 1A,B). The size of the gap, sometimes termed a compliance gap, has been used by some researchers to argue that catchments should be categorised as those that should be prioritised for either protection, agricultural production or nutrient level mitigation, based on their amenity or agricultural potential relative to nutrient thresholds^60^. However, this must be tempered by what can be achieved on farm – which requires consideration of economic, soil and cultural factors as well as lag times in determining the timing of, and degree to which the benefits of action are realised^31^. If the likelihood of achieving an objective through changing practices is poor, land use may have to change, in which case the requirements for achieving an objective will be best determined by land-use suitability assessments^61^. It is also worth noting that nutrient enrichment may also cause other negative influences on biotic integrity, especially in oligotrophic streams where nutrient concentrations are low^62^. Our models may therefore aid in protecting these vulnerable streams before undesirable periphyton growth is noted.

Unlike rivers, algal growth in estuaries and coastal waters is generally found to be N-limited^63^. Analyses at a continental and global level suggest that N loss from the land to estuaries and coastal waters is likely to increase by 2050 (e.g., by 43% in Africa)^64^. Although some work has also occurred at a country level looking at ratios of N to P loss (e.g., in China)^65^, finer scale data are required to match changes in populations, land use and the effect of climate change on nutrient loss pathways and profiles within catchments^66^. Our data may therefore be used to indicate the location of coastal areas that may be at risk of increased nutrient loss from the land to coastal waters.

## Conclusions

We predict that 31% of the global land area contains catchments with larger rivers (order 6 and above) exhibit undesirable levels of periphyton growth. Eight of the 31% was caused by enrichment with growth limited by N impacting 280 M people, while the remainder was caused by enrichment with growth limited by P impacting on 1.7B people. Much of this P-enrichment was in heavily populated catchments dominated by agricultural land in North and South America and Europe, but also in sparsely populated areas in Asia prone to erosion. Much of the N-enrichment likely to cause undesirable periphyton growth was in areas of North Africa, and parts of the Middle East and India. These findings can be used by landowners and policy makers to better target investment and actions at finer scales, to remediate poor water quality and aquatic habitats impaired by undesirable levels of periphyton growth.

## Materials and Methods

### Database sources, harmonisation and filtering

We started by identifying published datasets for site-specific nutrient concentrations in streams and rivers according to their global coverage. Each of these datasets published standards for recording (see references in Supplementary Table S5 for data standards). The raw data are available at https://doi.org/10.6084/m9.figshare.11555844. Note that use of these data should cite those data sources in Supplementary Table S5. However, we used a multi-step harmonisation process described by Larned, *et al*.^67^ to produce one global dataset that used consistent units, and laboratory detection and reporting limits. These steps comprised:

1. Plotting time-series to identify and correct data errors, such as values carrying incorrect units.
2. Imputing replacement values for data below the detection limit. Replacements were made at half the detection limit for the site and to no more than 10% of data points for a site. If >10% of data were below the detection limit, the site was excluded from further processing. Sites with >10% of the data below the detection limit represented <2% of total sites in the initial dataset. Although these could be largely oligotrophic sites the small number of sites excluded were not expected to bias the models.
3. Checking that analytical methods were acceptable. We accepted TN and TP concentrations generated via the digestion of unfiltered samples in strong acid (e.g. HCl, HNO_3_) or acidified-persulfate. For dissolved reactive P (DRP), we accepted samples filtered <0.7 µm. Measurements of P based on molybdenum blue colorimetry or ion chromatography were considered comparable. Measurements of nitrate or nitrite-N, which were obtained using ion chromatography, cadmium reduction, azo dye colorimetry or optical sensor methods, were presumed to be comparable^68^, whereas measurements obtained using all other methods were omitted.

For each variable, we examined data collected between 1990 and 2016. We focused our efforts on the most recent seven years of data collected at baseflow. We matched concentrations sites to sites where discharge was measured on a daily basis at a site in the catchment that was no more than 50 km downstream^69^. Catchments were defined at the fourth level of HydroBASINS^70^, which delineates 6020 catchments globally. We separated discharge into baseflow and stormflow using a recursive digital filter^71^ and only included data that contained a minimum of 75% of baseflow. This percentile avoided data taken during stormflow where periphyton was likely to be swept away or growth was likely inhibited by suspended sediment and light limitation^5^. We excluded monitoring sites for which there were <37 data points and were sampled less frequently than at least once every two months. This exclusion filter resulted in a dataset whose mean sampling age was 2008. Our decision to use data from sites with a minimum sampling number of 37 and that were sampled between the defined date range was a trade-off between obtaining enough sites to reflect wide spatial coverage, and that these sites contained enough samples to not affect the accuracy of median concentrations^72^. The seven-year period also help us avoid some variations associated with continental or global climatic trends. For example, in the Southern Hemisphere, the Southern Oscillation Index results in distinct trends in nutrient concentrations associated with wet and dry years^73^. The seven-year period also minimises, but does not exclude, any likely influence from long-term anthropogenic trends due to factors such as changes in land use and land use practices^67^.

We focused our data to periods of likely peak growth by filtering data for temperate and polar regions (i.e., regions outside the tropics of Cancer and Capricorn) to values measured from May to October in the Northern Hemisphere and for November to April in the Southern Hemisphere. All data were used to generate medians for tropical regions.

Predictor variables were chosen to reflect categorical and continuous factors known to affect N and P losses to water, such as land use and land use intensity^26,74^, and catchment and climate characteristics^75^. Continuous variables (e.g., percentage cropland) were expressed as a mean across a catchment. Where more than one categorical variable (e.g., ecoregion) was present in a catchment, the catchment was categorised as the category which covered the most of the catchment’s area. Predictor variables were obtained from a wide variety of sources and are listed in the Supplementary Table S6).

The number of catchments was reduced by filtering out those catchments for which a full set of predictor variables could not be found. After harmonising, pairing and filtering all datasets, the final filtered dataset that was used for modelling contained a total of 566,024 concentration measurements for DRP, TP, NO3-N and TN at 837, 1074, 897 and 793 sites, respectively (Supplementary Table S5; Fig. S3). Not all nutrient forms were measured at each site resulting in data for 1406 unique catchments.

### Estimation of global median concentrations and likely periphyton growth

The nutrient concentrations in our harmonised dataset were skewed by sites exhibiting enrichment. A Shapiro-Wilk test indicated that the log-transformation resulted in a normally distributed dataset.

Variables were included in a multiple linear regression using a best subsets routine in the R-programming language (www.r-project.org). An optimal regression output (i.e., avoiding over-parameterisation) was generated with the aid of the Mallows Cp statistic. Predicted concentrations were back-transformed and corrected for retransformation bias using the smearing estimate^76^:

1 $$S=\frac{1}{n}\mathop{\sum }\limits_{i=1}^{n}{e}^{\widehat{{\varepsilon }_{i}}}$$

where ${\hat{\varepsilon }}_{i}$ represents the residuals of the regression models. The correction factor (*S*) was applied over the whole range of predictions because it was assumed that the residuals were homoscedastic.

To validate the models, we used the Murray-Darling database (23 catchments with 22,374 concentrations measurements) and a subset of 20 catchments data (with 11,612 concentration measurements) from the New Zealand Water Quality database. We removed these sites from the combined database to maintain their independence from the data used to generate the global model (Supplementary Fig. S3). We selected only catchments that contained Strahler stream orders 6 or above because these approximated the mean stream order of 6.6 for catchments in the global database. For the NZWQ database this represented 28% of rivers of Strahler stream order 6 or greater. The catchments were also chosen because they represented a wide range of temperate and tropical biomes and include deserts and xeric shrublands. However, they do not include catchments in tundra regions.

The validated models were used to predict median concentrations of DRP, NO_3_-N, TP and TN globally in ArcGIS (v10.1, Redlands, CA). Raster grids were created with a spatial resolution of 0.025 degrees, which corresponded to the coarsest grid cell associated with the input data listed in Supplementary Table S6. To provide consistent global coverage, grid values were averaged within the boundaries of the 283 catchments represented at HydroBASINS level 3 (all stream order 6 or greater)^70^. Catchments were assigned to continents based on which continent most of the catchment area occupied.

Predicted median concentrations within HydroBASINS level 3 catchments were also used to generate estimates of N or P limitation, and the likelihood of periphyton growth based on the concentration of the limiting nutrient. To estimate when and where the likelihood of periphyton growth above an undesirable level was likely to occur, we used nutrient threshold criteria. We surveyed the literature to select an appropriate threshold in the concentrations of nutrients (NO_3_-N, TN, DRP and TP), above which periphyton growth, detected as biomass or the concentration of chlorophyll-a, is deemed undesirable. Of the 27 studies identified in the literature survey, most were carried out in the US (15), with four covering streams and rivers in China and the rest performed in Europe, South America, Africa and Australasia (Supplementary Table S7). The most common N and P fraction measured globally was TN and TP. The mean threshold above which undesirable periphyton growth has been reported to occur for TN was 0.800 mg L^−1^ (S.E. = 0.073 mg L^−1^), ranging from 0.210 mg L^−1^ in Montane Cordillera, Canada to 2.010 mg L^−1^ in Pennsylvania, US. The mean threshold for TP was 0.046 mg L^−1^ (S.E. = 0.010 mg L^−1^), ranging from 0.010 mg L^−1^ in Norway and Sao Paulo State, Brazil to 0.155 mg L^−1^ in Washington and Nebraska, US. Six studies assessed relationships between periphyton growth and dissolved nutrient concentrations^48,77–81^. However, because there were only six studies, covering little of the globe, mean dissolved nutrient thresholds were not used in the prediction of periphyton growth.

Based on the Redfield N:P ratio of 7:1 (by mass) and the mean thresholds for TN and TP derived from the literature survey, each catchment was assessed to determine to which of four different catchment types the catchment belonged:

- If the N:P (as TN and TP) ratio is <7 and the median TN concentration is <0.800 mg L^−1^, the site will have a quantity of periphyton growth that is acceptable and is N-limited (catchment type 1).
- If the N:P (as TN and TP) ratio is <7 and the median TN concentration is >0.800 mg L^−1^, the site will have a quantity of periphyton growth that is undesirable and is N-limited (catchment type 2).
- If the N:P (as TN and TP) ratio is ≥7 and the median TP concentration is <0.046 mg L^−1^, the site will have a quantity of periphyton growth that is acceptable and is P-limited (catchment type 3).
- If the N:P (as TN and TP) ratio is ≥7 and the median TP concentration is >0.046 mg L^−1^, the site will have a quantity of periphyton growth that is undesirable and is P-limited (catchment type 4).

Although we chose to restrict our estimation of nutrient thresholds to those required to prevent undesirable levels of periphyton growth, N and P thresholds of a similar magnitude were set to prevent the undesirable growth of other primary producers in lotic systems. For example, thresholds of 0.270–0.600 mg TN L^−1^ and 0.022–0.044 mg TP L^−1^ have been established for various US lakes to ensure that there is a low risk of cyanobacteria bloom, and thresholds of 0.800–1.400 mg TN L^−1^ and 0.061–0.250 mg TP L^−1^ have been set for a moderate risk of cyanobacteria bloom^82^.

Literature thresholds were further classified into a range of temperate and tropical biomes (Supplementary Table S7). However, a one-way analysis of variance showed that threshold means were no different across biomes. We therefore decided to use mean thresholds of 0.800 mg L^−1^ and 0.046 mg L^−1^ for N and P, respectively, to indicate undesirable periphyton growth in rivers in the global assessment.

## Supplementary information

Supplementary information.
