# Supplementary material for: Global mapping of freshwater nutrient enrichment and periphyton growth potential
Source: Sci Rep. 2020 Feb 27;10:3568. doi: 10.1038/s41598-020-60279-w (PMC7046692; doi:10.1038/s41598-020-60279-w)
Supplement: Supplementary file 1 — Supplementary information. [file 41598_2020_60279_MOESM1_ESM.pdf]

Supplementary Information:

Global mapping of freshwater nutrient enrichment and periphyton growth potential

R. W. McDowell[1,2,*], A. Noble[1], P. Pletnyakov[1], B. Haggard[3], L. M. Mosley[4]

[1]*AgResearch, Lincoln Science Centre, Private Bag 4749, Christchurch 8140, New Zealand.*

[2]*Faculty of Agriculture and Life Sciences, P O Box 84, Lincoln University, Lincoln 7647, Christchurch, New Zealand.*

[3]*Biological and Agricultural Engineering Department, University of Arkansas, Fayetteville, AR 72703*

[4]*School of Biological Sciences, University of Adelaide, SA 5005 Australia*

[*]Corresponding author's email: Richard.mcdowell@agresearch.co.nz

Table S1. Coefficients, standard errors and levels of significance used to predict global dissolved reactive P concentrations depending on the stated variables. Biomes refer to US EPA Ecoregions [15]. Log-transformed data must be multiplied by the bias correction factor after back-transformation.

Variable	Estimate	Standard error	P value
Intercept	-7.5078	0.5265	<0.001
Olsen P (kg ha^{-1})	0.0268	0.0030	<0.001
Mean precipitation (mm)	-0.0104	0.0026	<0.001
Mean slope (%)	-0.0728	0.0329	0.027
Biome - deserts & xeric shrublands	2.4167	0.5039	<0.001
Biome - Mediterranean forests, woodlands & scrub	2.7699	0.5766	<0.001
Biome - montane grasslands & shrublands	1.6239	0.5457	0.003
Biome - temperate broadleaf & mixed forests	1.5045	0.4426	0.001
Biome - temperate conifer forests	2.3976	0.4844	<0.001
Biome - temperate grasslands, savannas & shrublands	2.5965	0.4579	<0.001
Biome - tropical & subtropical dry broadleaf forests	0.4863	0.5874	0.408
Biome - tropical & subtropical grasslands, savannas & shrublands	2.1517	0.5455	<0.001
Biome - tropical & subtropical moist broadleaf forests	1.6839	0.5246	0.001
Biome - tundra	3.2534	0.9481	0.001
Potential evapotranspiration (mm)	0.0182	0.0047	<0.001
Soil wetness (mm over profile)	0.0067	0.0029	0.021
Population density (2000)	0.0032	0.0005	<0.001
Coefficient of determination	0.35		
Bias correction	1.290		

Table S2. Coefficients, standard errors and levels of significance used to predict global total P concentrations depending on the stated variables. Biomes refer to US EPA Ecoregions [15]. Log-transformed data must be multiplied by the bias correction factor after back-transformation.

Variable	Estimate	Standard error	P value
Intercept	-4.2396	0.2161	<0.001
Olsen P (kg ha^{-1})	0.0100	0.0017	<0.001
Mean precipitation (mm)	-0.0062	0.0010	<0.001
Mean slope (%)	-0.0768	0.0198	<0.001
Biome - deserts & xeric shrublands	1.4481	0.2496	<0.001
Biome - Mediterranean forests, woodlands & scrub	1.8352	0.3854	<0.001
Biome - montane grasslands & shrublands	0.5575	0.2531	0.028
Biome - temperate broadleaf & mixed forests	0.5216	0.1902	0.006
Biome - temperate conifer forests	0.8749	0.2220	<0.001
Biome - temperate grasslands, savannas & shrublands	1.0591	0.1990	<0.001
Biome - tropical & subtropical dry broadleaf forests	0.2002	0.3356	0.551
Biome - tropical & subtropical grasslands, savannas & shrublands	0.9004	0.2549	<0.001
Biome - tropical & subtropical moist broadleaf forests	1.0789	0.2381	<0.001
Biome - tundra	1.9464	0.4573	<0.001
Cropland (%)	0.0075	0.0011	<0.001
Potential evapotranspiration (mm)	0.0134	0.0029	<0.001

Coefficient of determination	0.41
Bias correction	0.855

Table S3. Coefficients, standard errors and levels of significance used to predict global nitrate-nitrite-N concentrations depending on the stated variables. Biomes refer to US EPA Ecoregions [15]. Log-transformed data must be multiplied by the bias correction factor after back-transformation.

Variable	Estimate	Standard error	P value
Intercept	-4.8564	0.3595	<0.001
Biome - deserts & xeric shrublands	2.0325	0.3973	<0.001
Biome - Mediterranean forests, woodlands & scrub	1.3059	0.4883	0.008
Biome - montane grasslands & shrublands	0.2563	0.5353	0.632
Biome - temperate broadleaf & mixed forests	1.5984	0.3358	<0.001
Biome - temperate conifer forests	1.3921	0.3680	<0.001
Biome - temperate grasslands, savannas & shrublands	1.8649	0.3320	<0.001
Biome - tropical & subtropical dry broadleaf forests	2.0295	0.9102	0.026
Biome - tropical & subtropical grasslands, savannas & shrublands	1.0883	0.3853	0.005
Biome - tropical & subtropical moist broadleaf forests	1.4408	0.3565	<0.001
Biome - tundra	0.1286	1.2705	0.919
Population density (2000)	0.0358	0.0107	0.001
Soil wetness (mm over profile)	0.0198	0.0022	<0.001
Area (km^2)	0.0001	0.0000	0.001
Olsen P ($kg\ ha^{-1}$)	0.0279	0.0046	<0.001
Population density (1995)	-0.0342	0.0110	0.002
Lentic (%)	-0.1808	0.0455	<0.001
Forest (%)	-0.0225	0.0022	<0.001
Mean slope (%)	0.0203	0.0342	0.552
Coefficient of determination	0.49		
Bias correction	1.440		

Table S4. Coefficients, standard errors and levels of significance used to predict global total N concentrations depending on the stated variables. Biomes refer to US EPA Ecoregions [15]. Log-transformed data must be multiplied by the bias correction factor after back-transformation.

Variable	Estimate	Standard error	P value
Intercept	-1.5181	0.2136	<0.001
Mean precipitation (mm)	-0.0089	0.0012	<0.001
Mean slope (%)	-0.1838	0.0193	<0.001
Biome - deserts & xeric shrublands	1.9935	0.2435	<0.001
Biome - Mediterranean forests, woodlands & scrub	3.2782	0.4312	<0.001
Biome - montane grasslands & shrublands	1.5238	0.2471	<0.001
Biome - temperate broadleaf & mixed forests	1.3245	0.1829	<0.001
Biome - temperate conifer forests	1.8488	0.2202	<0.001
Biome - temperate grasslands, savannas & shrublands	1.6756	0.1891	<0.001
Biome - tropical & subtropical dry broadleaf forests	1.0801	0.2509	<0.001

Biome - tropical & subtropical grasslands, savannas & shrublands	0.5203	0.2309	0.025
Biome - tropical & subtropical moist broadleaf forests	1.2144	0.2089	<0.001
Cropland (%)	0.0106	0.0011	<0.001
Soil wetness (mm over profile)	0.0055	0.0015	<0.001
Coefficient of determination	0.60		
Bias correction	0.595		

Table S5. Data sources and the number of catchments and data records remaining after harmonisation and filtering. These harmonised and filtered data were used to predict and validate the median concentrations of phosphorus and nitrogen forms.

Database/reference	N or P fraction	Number of catchments	Number of data records	Number of catchments following harmonisation and filtering	Mean number of harmonised and filtered data records
GEMStat [1]	DRP	215	53,703	106	27,217
	TP	278	109,398	249	38,209
	NO_3-N	674	68,634	130	22,262
	TN	777	32,558	68	12,030
GLORICH [2]	DRP	11,835	660,942	394	54,017
	TP	10,532	484,700	488	89.484
	NO_3-N	7,401	583,114	430	80,021
	TN	4,685	267,060	388	60,887
Murray-Darling [3]	DRP	23	34,642	23	5,431
	TP	23	35,889	23	5,715
	NO_3-N	23	34,454	23	5,559
	TN	23	42,770	23	5,669
NZWQ [4,5]	DRP	728	74,573	357 (20)[1]	45,474 (2903)
	TP	728	74,571	357 (20)	45,474 (2903)
	NO_3-N	728	74,573	357 (20)	45,474 (2903)
	TN	728	74,573	357 (20)	45,474 (2903)

[1] Denotes the number of catchments and data used for validation purposes.

Table S6. List of units and sources of variables used to predict the concentrations of nitrogen and phosphorus.

Variable (years of data used)	Units	Source
Catchment area (2008)	km^2	[6]
Mean altitude (2008)	m above sea level	[6]
Mean slope (2008)	%	[6]
Net primary production - 2015	g C m^{-2} yr^{-1}	[7]
Soil order (2015)	% of catchment	[8]
Soil organic carbon (1950-1999)	g C m^{-2} yr^{-1}	[9]
Soil Olsen P stock (2010)	kg ha^{-1}	See Supplementary information 'Global Soil Map'
Mean potential evapotranspiration (1950-1999)	mm yr^{-1}	[9]
Soil wetness (1950-1999)	mm over profile	[9]
Discharge (2000)	m^3 yr^{-1}	[10]
Mean runoff by month (1-12) (2000)	mm	[10]
Mean rainfall (1970-2000)	mm yr^{-1}	[11]
Mean precipitation by month (1-12) (1970-2000)	mm	[11]
Crop cover 2009	% of catchment	[12]
Forest cover 2009	% of catchment	[12]
Lentic cover 2009	% of catchment	[12]
Pasture cover 2009	% of catchment	[12]
Rangeland cover 2009	% of catchment	[12]
Urban cover 2009	% of catchment	[12]
GDP per capita (2015)	USD $person^{-1}$	[13]
Population density (1990)	Number of people km^{-2}	[14]
Population density (1995)	Number of people km^{-2}	[14]
Population density (2000)	Number of people km^{-2}	[14]
Terrestrial biomes	Categorical	[15]
Ecoregion within biomes	Categorical	[15]
Global ecological land units - Bioclimate	Categorical	[16]
Global ecological land units – landform	Categorical	[16]
Global ecological land units - lithology	Categorical	[16]
Global ecological land units – land cover	Categorical	[16]

Table S7. Range and form of nitrogen and phosphorus thresholds designed to prevent unwanted periphyton blooms in streams and rivers. Data for selected biomes were compared using a one-way analysis of variance.

Location	Terrestrial biome	Threshold	Total N (nitrate-N), mg L^{-1}	Total P (dissolved reactive P), mg L^{-1}	Reference
US studies					
Arkansas and Oklahoma	Temperate broadleaf and mixed forests; Temperate grasslands, savannas and shrublands	Thresholds derived to protect from nuisance algal biomass	-	0.035	[17]
		Phosphorus thresholds based on the shift in periphyton biomass	-	0.018-0.040[1]	
		Phosphorus thresholds based on the proportion and biovolume of nuisance algal biomass	-	0.032-0.058	
		Phosphorus thresholds based on mean community level shifts (that is, species declining or increasing)	-	0.011-0.049	
Connecticut	Temperate broadleaf and mixed forests	Ranging from the protection of sensitive taxa to where tolerant diatoms increased	-	0.020-0.082	[18]
Florida	Temperate grassland, savannas and shrublands	Significant increase in macroalgae - *Lyngbya wollei* and *Vaucheria* spp.	0.250-0.284 (0.230-0.261)	0.026-0.033 (0.022-0.028)	[19]
Michigan and Kentucky	Temperate broadleaf and mixed forests	Significant response indicated by *Cladophora* coverage and chlorophyll-a concentrations	0.400-1.000	0.010-0.030	[20]
Mid-Atlantic Highlands, NE USA	Temperate broadleaf and mixed forests	Decreases in native taxa, especially diatoms	0.400	0.010-0.012	[21]
Minnesota	Temperate broadleaf and mixed forests; Temperate grasslands, savannas and shrublands	Recommended regional river eutrophication criteria	-	0.050-0.150	[22]
Montana	Temperate grassland, savannas and shrublands	Chlorophyll-a < 100 mg m^{-2} in streams	0.350	0.030	[23]
New Jersey	Temperate broadleaf and mixed forests	Change in impairment based on biological condition gradient in diatom assemblages	1.000	0.050	[24]
New Jersey and surrounding Atlantic states	Temperate broadleaf and mixed forests;	Change in impairment based on biological condition gradient in diatom assemblages		0.050	[25]

	Temperate grassland, savannas and shrublands				
New York	Temperate broadleaf and mixed forests	Protection of aquatic life as measured by chlorophyll-a, diatoms and macroinvertebrates	0.700 (0.300)	0.030	26
Ohio	Temperate broadleaf and mixed forests	Chlorophyll-a > 182 mg m^{-2}	(435)	0.038	27
Ohio	Temperate broadleaf and mixed forests	Sensitive taxa are lost and algal assemblage changes	-	0.040	18
Pennsylvania	Temperate broadleaf and mixed forests	Mid-point between impaired and unimpaired catchments for biological health	2.010	0.070	28
Various American states	Various	Change in trophic status of streams when chlorophyll-a > 100 mg m^{-2}	0.285-0.375	0.023-0.029	29
Washington, Nebraska	Temperate broadleaf and mixed forests	Range of thresholds to deter the growth of motile algae and diatoms	0.590-1.790	0.030-0.280	30
Wisconsin	Temperate broadleaf and mixed forests; Temperate grasslands, savannas and shrublands	Nutrient concentrations based on change in chlorophyll-a and diatom indices in wadeable streams	0.872-1.169	0.039-0.074	31
Other studies					
Sao Paulo state, Brazil	Tropical and subtropical moist broadleaf forests; Tropical and subtropical grasslands, savannas and shrublands	Oligotrophic (chlorophyll-a < 1.7 µg L^{-1})<br>Mesotrophic (chlorophyll-a < 9.0 µg L^{-1})	0.460<br>0.820	0.010<br>0.030	32
Atlantic maritime, Canada	Temperate broadleaf and mixed forests	Combination of measures to control benthic algae and protect macroinvertebrates	0.870-1.200	0.010-0.030	33
Montane Cordillera, Canada	Temperate broadleaf and mixed forests		0.210	0.020	
Mixed wood plains, Canada	Temperate broadleaf and mixed forests		1.100	0.030	
Interior prairies, Canada	Temperate grassland, savannas and shrublands		0.390-0.980	0.100	

Liao River Basin, China	Temperate broadleaf and mixed forests	Recommended criteria	1.000	0.040	34
		Chemical and biological thresholds related to human activity and water quality	0.750-1.288	0.035-0.101	
Streams of the Lake Taihu catchment, China	Temperate broadleaf and mixed forests	Preventing the growth of *Microcystis* in the lake by setting in-stream thresholds	0.800	0.050	35
Streams of the Lake Dianchi catchment, China	Tropical and subtropical moist broadleaf forests	Ranging from the maintenance of high-quality streams to preventing the nuisance growth of tolerant taxa	0.380-1.390	0.020-0.040	36
Streams of the Miyun Reservoir catchment, China	Temperate broadleaf and mixed forests	Designed to keep chlorophyll-a concentrations < 20 mg m^{-2} in reservoir	(0.500)	(0.040)	37
New Zealand	Temperate broadleaf and mixed forests, Temperate grassland, savannas and shrublands	Designed to keep chlorophyll-a < 120 mg m^2 in upland and lowland streams	0.614 (0.444)	0.033 (0.010)	38,39
	Montane grasslands and shrublands		0.295 (0.167)	0.026 (0.009)	
New Zealand	Temperate broadleaf and mixed forests	Declines in the most sensitive taxa	(0.070)	-	40
Norway	Temperate conifer forests, tundra	Responses of benthic algae to TP inputs	-	0.010-0.030	41
Touw and Duiwe River, South Africa	Mediterranean forests, woodlands and scrub	Suggested guidelines for total nutrients	0.252	0.035	42
Mean (temperate broadleaf and mixed forests) TN, TP only			0.914	0.046	
Mean (temperate grassland, savannas and shrublands) TN, TP only			0.732	0.058	
Mean (tropical biomes) TN, TP only			0.721	0.023	
Mean (overall)			0.800 (0.309)	0.046 (0.025)	
Standard error of the mean			0.073	0.010	
P value (biome)			0.300	0.576	

[1] For those studies exhibiting a range of thresholds, the mid-point is used to enable the calculation of an overall mean. This was thought to be the best compromise between studies that derived a single threshold for large catchments and those that derived multiple thresholds for smaller catchments.

A

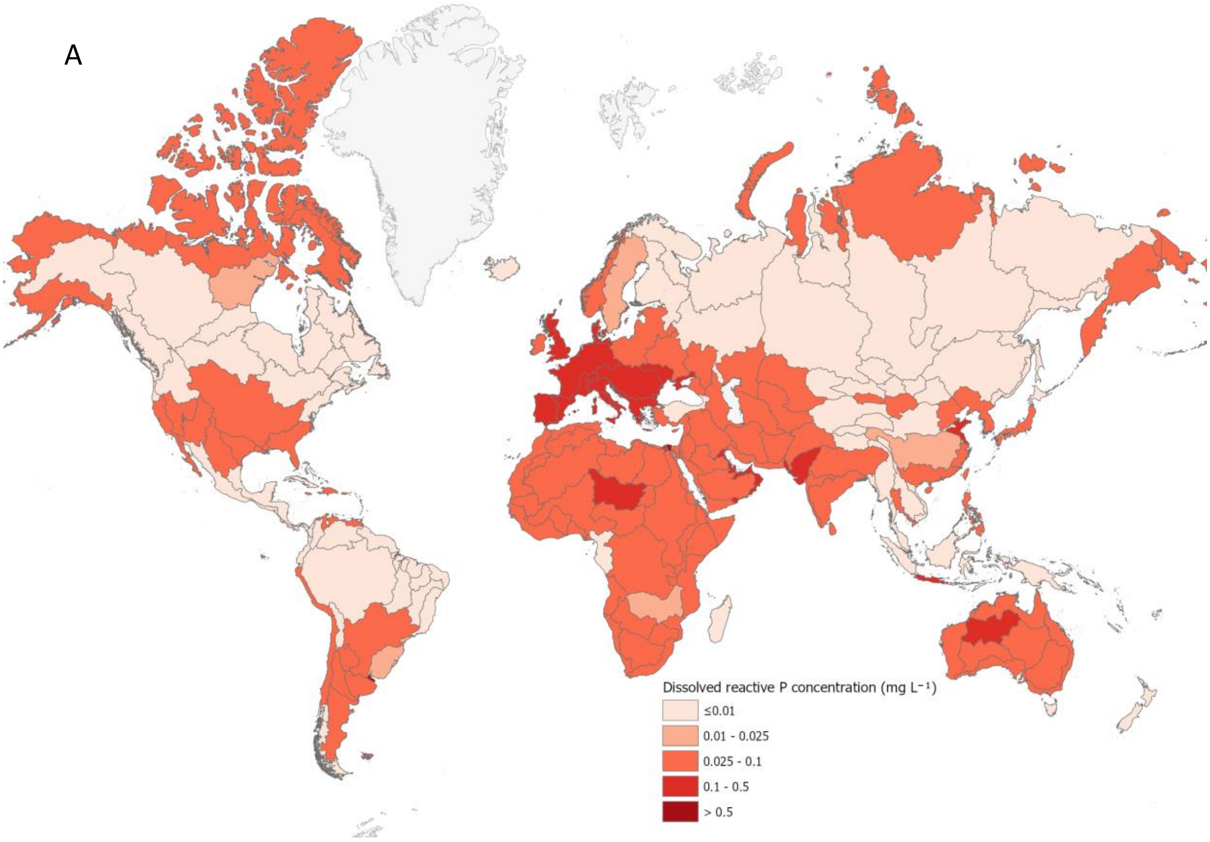

B

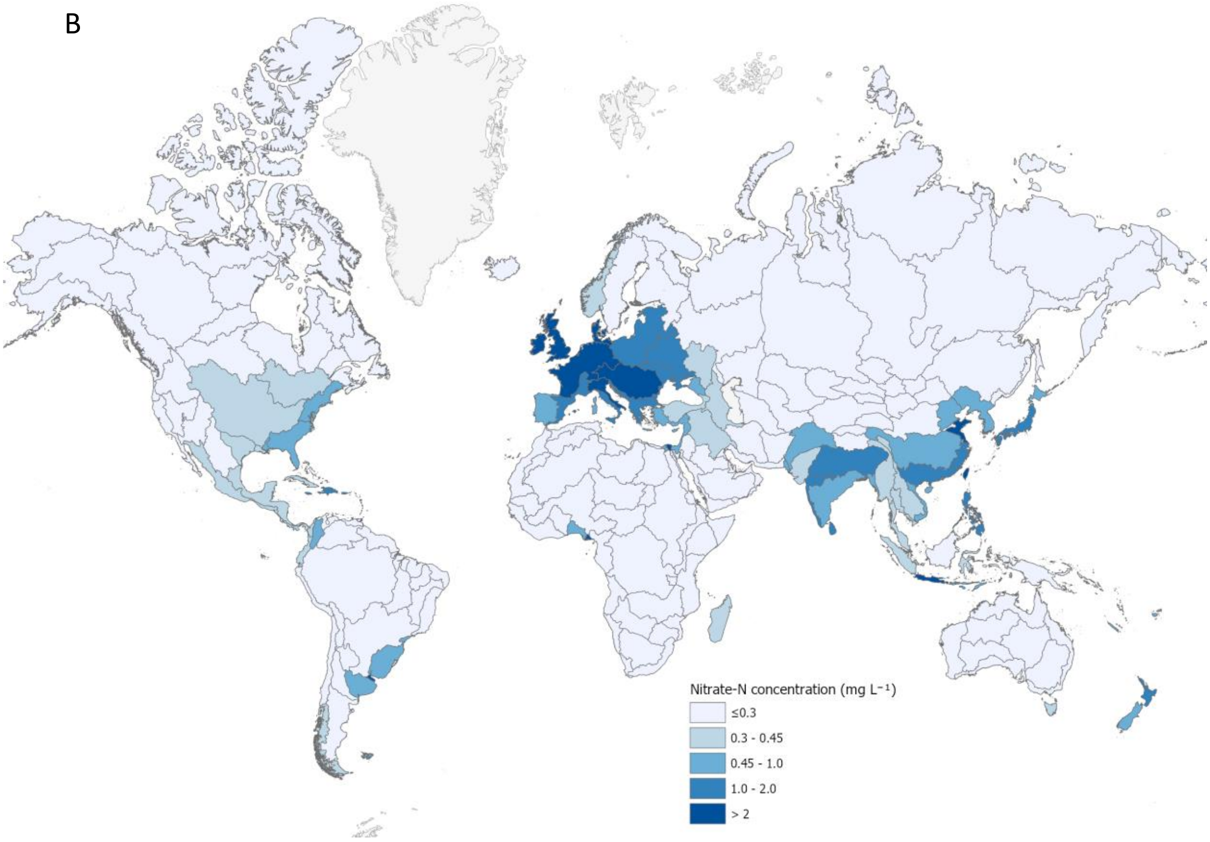

Figure S1A-B. Relative modelled median dissolved reactive P (A) and nitrate-N (B) concentrations (mg L^{-1}) during likely periphyton growth periods at catchment scale across the globe. Areas without

predictions are white (e.g., Greenland and Antarctica). The relative median values for each of the nutrients are depicted as five different concentration ranges, which include the threshold and half threshold concentration ranges.

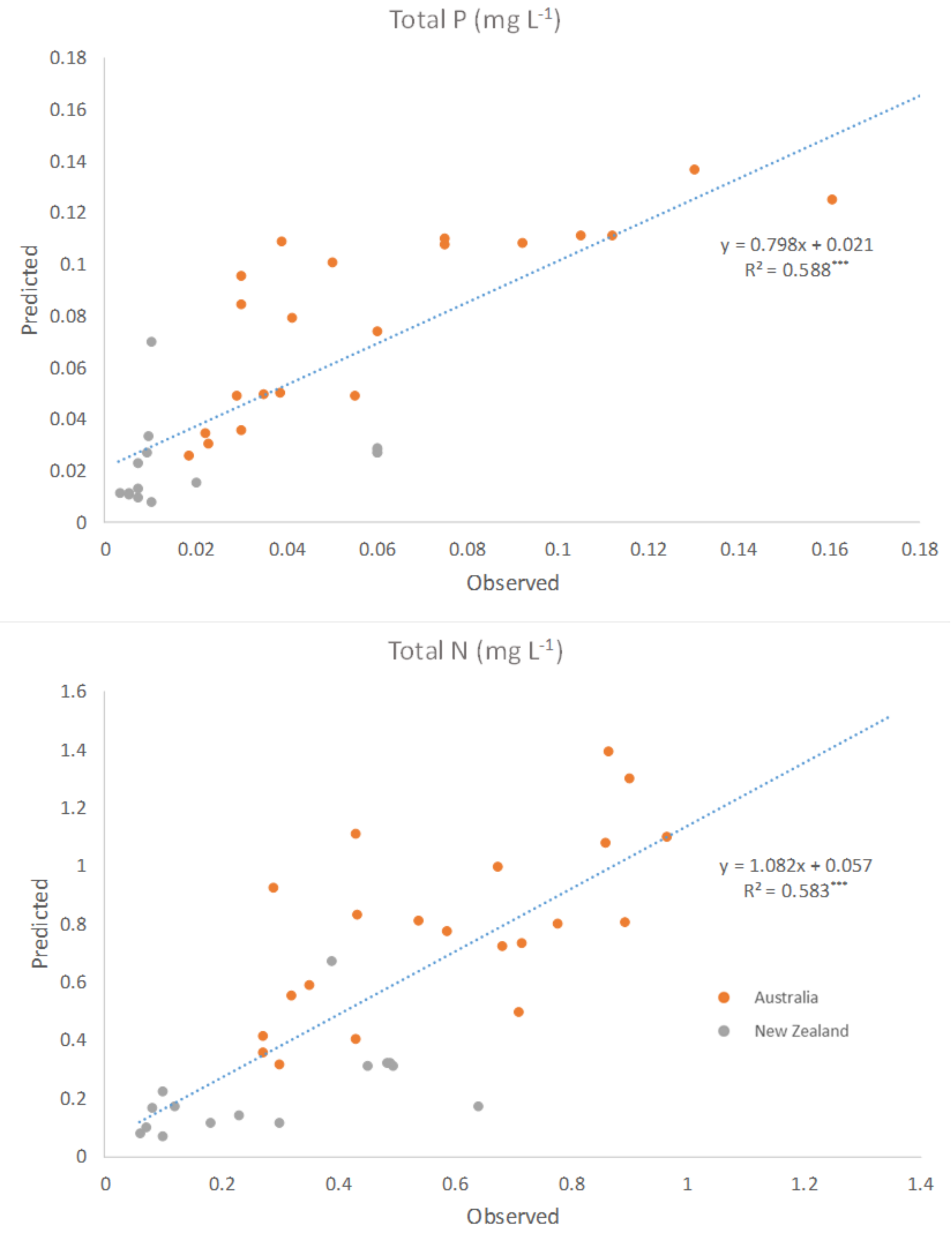

Figure S2. Relationships between predicted and observed total P (top) and N (bottom) concentrations for Australian and New Zealand validation catchments (stream order >6, n = 41, both relationships were significant at the *P*<0.001 level).

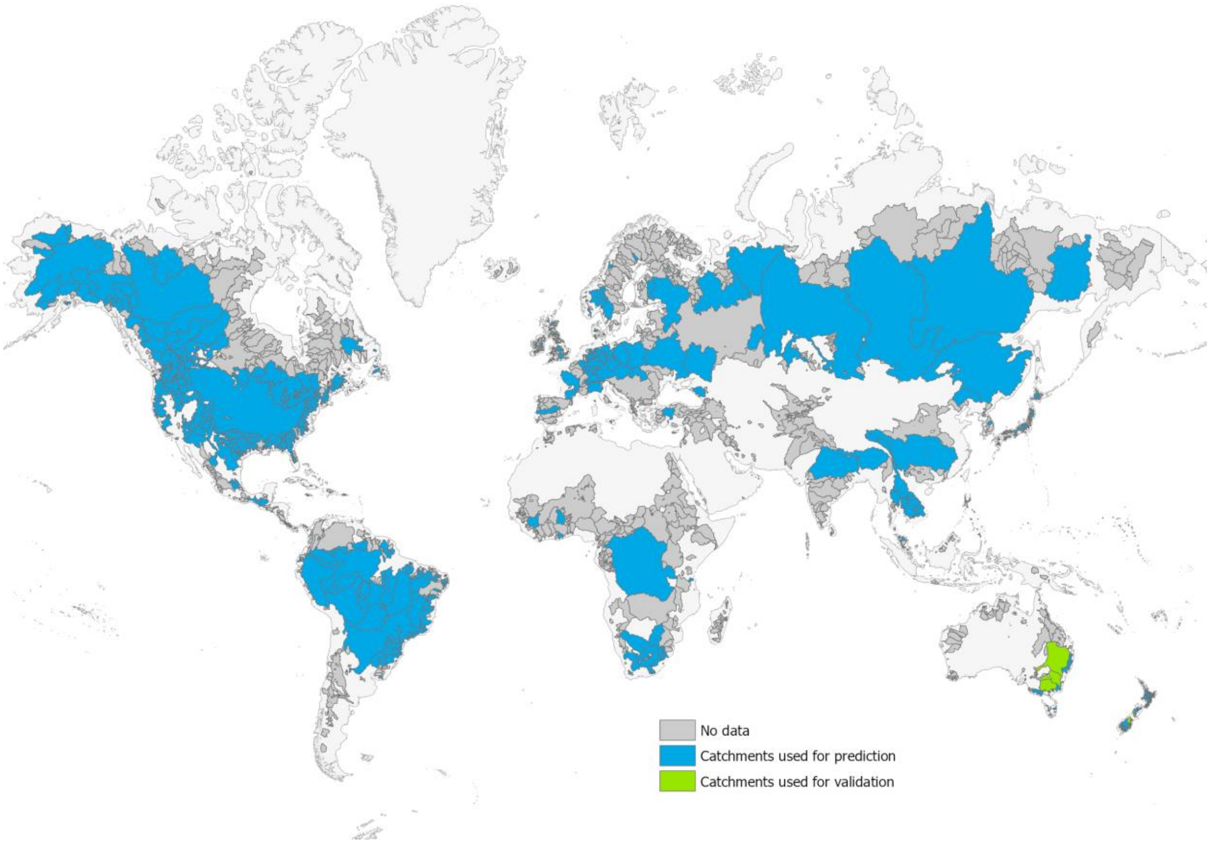

Figure S3. Catchment boundaries for all rivers whose data were used in the calculation (blue; n = 1406) and validation (green; n = 43) phases of model development. All other global catchments are coloured light grey (n = 6020).

Global map of soil Olsen phosphorus

Table S8. List of data sources used to construct the map shown in Fig. S4 of estimated global soil Olsen P for 2009-2011.

Data source	Number of samples	Year	Conversion to Olsen P	Coverage	References
ISRIC-World Soil Information	23,538	2010	20900 samples converted from Bray-I P or Mehlich III using Malo and Gelderman [43] for soils > pH 6 and using Wolf and Baker [44] for soils ≤ pH 6; conversion was not necessary for 2638 samples	Global	[45]
LUCAS Topsoil Survey	19,965	2009	Conversion unnecessary – already in Olsen P	Europe	[46]
ASRIS	6,537	2011	Converted from Colwell P using [47]	Australia	[48]
NZ Soil database	47,206	2011	Conversion unnecessary – already in Olsen P	New Zealand	[49]

Of the 97246 samples in the database, 69809 were measured as Olsen P. Published regression equations were used to convert other soil test P methods to Olsen P equivalents (Table S8). The ASRIS database also contained total P data for 7247 samples of topsoil collected at depths of 0-20 cm

Modelling of soil Olsen P

Information was gained for soil group, land cover, and gross domestic product per capita (GDPPC) from http://www.fao.org/soils-portal/soil-survey/soil-maps-and-databases/harmonized-world-soil-database-v12/en/ [50]. A full treatment combination of soil group by land cover by GDPPC for each country was fitted as random terms via a random effect, maximum likelihood analysis in Genstat [51], generating a mean estimated Olsen P concentration for each combination. Data were analysed on a log scale. We analysed 17906 combinations of countries (including GDPPC) by land cover and soil group to estimate a mean Olsen P concentration. Another 974 combinations, mostly relating to areas in north African countries, could not be modelled. These were assigned a default Olsen P value of 2 mg P/kg, equivalent to values found in undeveloped soils [52]. The model estimated 39% of the variation in the data.

Soil groups and land cover were converted to a 400-m equal area raster; each pixel therefore represents an area of 160,000 m^2. Soil Olsen P concentrations were assigned to the topsoil of grassland, forests and cropland (i.e., the top 7.5, 10 and 20 cm, respectively). United Nations Food and Agriculture Organization soil information [8] was used to assign a bulk density for each soil group in t m^{-3} (values equal g cm^{-3}) and the mass of soil in each pixel was calculated in kilotons. Assigned modelled Olsen P concentrations were used to calculate the tonnes of Olsen P in each pixel. The same process was used to calculate the mass of total P for the continent of Australia.

Results

The results of the modelling are shown for 256 ha parcels of land across the globe in Supplementary Figure S4. As a check of the model's performance, we compared our calculated mass of Olsen P for Africa to that estimated as Mehlich-3 P by Hengl, et al. [53] using WoSIS data. The mass of Mehlich-3 extractable P stored in the top 30 cm of soils for Sub-Saharan Africa was calculated to be 93,195 kt, which was 3.14 times more than our modelled estimate of 29636 kt as Olsen P. Nevertheless our modelled estimate was comparable to that of Hengl, et al. [53] given that Mehlich-3 extracts about three times more P than the Olsen method [44].

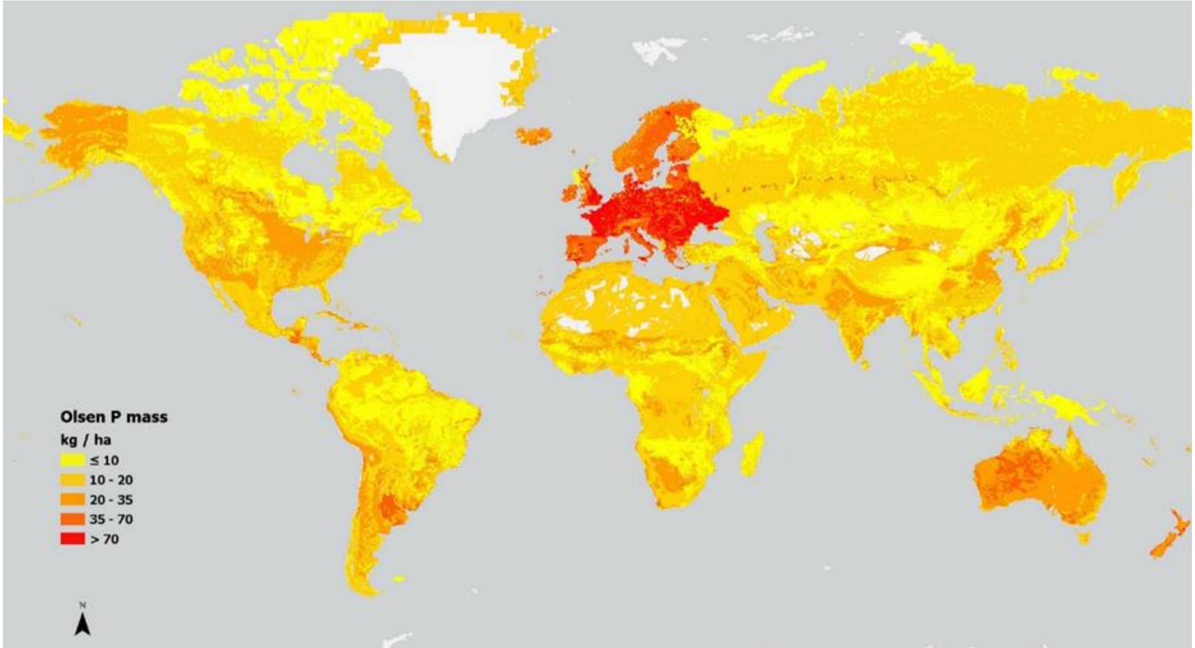

Figure S4. Raster map (256 ha resolution) showing the estimated mass (kg ha^{-1}) of soil Olsen P in the topsoil (top 7.5, 10 and 20-cm for grassland, forests and cropland, respectively). Areas without predictions are white.

References

1 International Centre for Global Water Resources and Global Change. *GEMStat*, <https://gemstat.org> (2018).

2 Hartmann, J., Lauerwald, R. & Moosdorf, N. A Brief Overview of the GLObal RIver Chemistry Database, GLORICH. *Procedia Earth and Planetary Science* **10**, 23-27, doi:https://doi.org/10.1016/j.proeps.2014.08.005 (2014).

3 Biswas, T. K. & Mosley, L. M. From Mountain Ranges to Sweeping Plains, in Droughts and Flooding Rains; River Murray Water Quality over the Last Four Decades. *Water Resour. Manage.*, doi:10.1007/s11269-018-2168-1 (2018).

4 McDowell, R. W., Snelder, T. H., Cox, N., Booker, D. J. & Wilcock, R. J. Establishment of reference or baseline conditions of chemical indicators in New Zealand streams and rivers relative to present conditions. *Mar. Freshwat. Res.* **64**, 387-400, doi:10.1071/mf12153 (2013).

5 Larned, S. T., Snelder, T., Unwin, M. J. & McBride, G. B. Water quality in New Zealand rivers: current state and trends. *N. Z. J. Mar. Freshwat. Res.* **50**, 1-29, doi:10.1080/00288330.2016.1150309 (2016).

6 U.S. Dept. of the Interior, U. S. G. S. *HydroSHEDS*, 2008).

7 NASA. *MODIS Gross Primary Production (GPP) / Net Primary Production (NPP)*, <https://modis.gsfc.nasa.gov/data/dataprod/mod17.php> (2018).

8 IUSS Working Group WRB. World Reference Base for Soil Resources 2014, update 2015 International soil classification system for naming soils and creating legends for maps. (Food and Agrculture Orgnisation, Rome, Italy, 2015).

9 Willmott, C. J. & Matsuura, K. *Terrestrial Air Temperature and Precipitation: Monthly and Annual Time Series (1950 - 1999)*, <http://climate.geog.udel.edu/~climate/html_pages/download.html> (2001).

10 Fekete, B. M., Vörösmarty, C. J. & Grabs, W. *UNH / GRDC Composite Runoff Fields v1.0*, <http://www.compositerunoff.sr.unh.edu/> (2018).

11 Fick, S. E. & Hijmans, R. J. WorldClim 2: new 1-km spatial resolution climate surfaces for global land areas. *International Journal of Climatology* **37**, 4302-4315, doi:doi:10.1002/joc.5086 (2017).
12 European Space Agency. *European Space Agency GlobCover Portal - GlobCover 2009*, <http://due.esrin.esa.int/page_globcover.php> (2010).
13 World Bank. *GDP per capita (current US$)*, <https://data.worldbank.org/indicator/NY.GDP.PCAP.CD?view=map> (2018).
14 Center for International Earth Science Information Network - CIESIN - Columbia University & Centro Internacional de Agricultura Tropical - CIAT. (NASA Socioeconomic Data and Applications Center (SEDAC), Palisades, NY, 2005).
15 Dinerstein, E. *et al.* An Ecoregion-Based Approach to Protecting Half the Terrestrial Realm. *Bioscience* **67**, 534-545, doi:10.1093/biosci/bix014 (2017).
16 Sayre, R. *et al.* A New Map of Global Ecological Land Units - An Ecophysiographic Stratification Approach. 46 (Association of American Geographers, Washington D.C., 2014).
17 King, R. S. Final Report to Governors from the Joint Study Committee and Scientific Professionals 70 (Scenic Rivers Joint Study Committee, Oklahoma Conservation Comission, 2016).
18 Smucker, N. J., Becker, M., Detenbeck, N. E. & Morrison, A. C. Using algal metrics and biomass to evaluate multiple ways of defining concentration-based nutrient criteria in streams and their ecological relevance. *Ecol. Indicators* **32**, 51-61, doi:https://doi.org/10.1016/j.ecolind.2013.03.018 (2013).
19 Stevenson, J. J., Pinowska, A., Albertin, A. & Sickman, J. O. Ecological condition of algae and nutrients in Florida Springs: The synthesis report. 58 (Department of Zoology, Michigan State University, East Landsing, MI, 2007).
20 Stevenson, R. J., Rier, S. T., Riseng, C. M., Schultz, R. E. & Wiley, M. J. Comparing Effects of Nutrients on Algal Biomass in Streams in Two Regions with Different Disturbance Regimes and with Applications for Developing Nutrient Criteria. *Hydrobiologia* **561**, 149-165, doi:10.1007/s10750-005-1611-5 (2006).
21 Stevenson, R. J., Hill, B. H., Herlihy, A. T., Yuan, L. L. & Norton, S. B. Algae–P relationships, thresholds, and frequency distributions guide nutrient criterion development. *J. N. Am. Benthol. Soc.* **27**, 783-799, doi:10.1899/07-077.1 (2008).
22 Heiskary, S. A. & Jr., R. W. B. Development of eutrophication criteria for Minnesota streams and rivers using multiple lines of evidence. *Freshwater Science* **34**, 574-592, doi:10.1086/680662 (2015).
23 Dodds, W. K., Smith, V. H. & Zander, B. Developing nutrient targets to control benthic chlorophyll levels in streams: A case study of the Clark Fork River. *Water Res.* **31**, 1738-1750, doi:http://dx.doi.org/10.1016/S0043-1354(96)00389-2 (1997).
24 Charles, D. F., Tuccillo, A. P. & Belton, T. J. Use of diatoms for developing nutrient criteria for rivers and streams: A Biological Condition Gradient approach. *Ecol. Indicators* **96**, 258-269, doi:https://doi.org/10.1016/j.ecolind.2018.08.048 (2019).
25 Hausmann, S., Charles, D. F., Gerritsen, J. & Belton, T. J. A diatom-based biological condition gradient (BCG) approach for assessing impairment and developing nutrient criteria for streams. *Sci. Total Environ.* **562**, 914-927, doi:https://doi.org/10.1016/j.scitotenv.2016.03.173 (2016).
26 Smith, A. J. & Tran, C. P. A weight-of-evidence approach to define nutrient criteria protective of aquatic life in large rivers. *J. N. Am. Benthol. Soc.* **29**, 875-891, doi:10.1899/09-076.1 (2010).
27 Miltner, R. J. A Method and Rationale for Deriving Nutrient Criteria for Small Rivers and Streams in Ohio. *Environ. Manage.* **45**, 842-855, doi:10.1007/s00267-010-9439-9 (2010).

28 Sheeder, S. A. & Evans, B. M. Estimating nutrient and sediment threshold criteria for biological impairment in Pennsylvania watersheds. *JAWRA Journal of the American Water Resources Association* **40**, 881-888, doi:doi:10.1111/j.1752-1688.2004.tb01052.x (2004).
29 Dodds, W. K. Eutrophication and trophic state in rivers and streams. *Limnol. Oceanogr.* **51**, 671-680, doi:doi:10.4319/lo.2006.51.1_part_2.0671 (2006).
30 Black, R. W., Moran, P. W. & Frankforter, J. D. Response of algal metrics to nutrients and physical factors and identification of nutrient thresholds in agricultural streams. *Environ. Monit. Assess.* **175**, 397-417, doi:10.1007/s10661-010-1539-8 (2011).
31 Robertson, D. M. *Nutrient concentrations and their relations to the biotic integrity of wadeable streams in Wisconsin*. 139 (U.S. Dept. of the Interior, U.S. Geological Survey, 2006).
32 Cunha, D. G. F., Ogura, A. P. & Calijuri, M. D. C. Nutrient reference concentrations and trophic state boundaries in subtropical reservoirs. *Water Science and Technology* **65**, 1461-1467, doi:10.2166/wst.2012.035 (2012).
33 Chambers, P. A. *et al.* Development of Environmental Thresholds for Nitrogen and Phosphorus in Streams. *J. Environ. Qual.* **41**, 7-20, doi:10.2134/jeq2010.0273 (2012).
34 Chen, J., Li, F., Wang, Y. & Kong, Y. Estimating the nutrient thresholds of a typical tributary in the Liao River basin, Northeast China. *Scientific reports* **8**, 3810, doi:10.1038/s41598-018-22128-9 (2018).
35 Xu, H. *et al.* Determining Critical Nutrient Thresholds Needed to Control Harmful Cyanobacterial Blooms in Eutrophic Lake Taihu, China. *Environ. Sci. Technol.* **49**, 1051-1059, doi:10.1021/es503744q (2015).
36 Cao, X. *et al.* Establishment of stream nutrient criteria by comparing reference conditions with ecological thresholds in a typical eutrophic lake basin. *Environmental Science: Processes & Impacts* **19**, 1554-1562, doi:10.1039/C7EM00074J (2017).
37 Zeng, Q., Qin, L., Bao, L., Li, Y. & Li, X. Critical nutrient thresholds needed to control eutrophication and synergistic interactions between phosphorus and different nitrogen sources. *Environmental Science and Pollution Research* **23**, 21008-21019, doi:10.1007/s11356-016-7321-x (2016).
38 Australian and New Zealand Environment and Conservation Council & Agriculture and Resource Management Council of Australia and New Zealand. Australiand and New Zealand Guidelines for Fresh and Marine Water Quality: Volume 2 Aquatic Ecosystems - Rationale and Background Information. 678 (Australian and New Zealand Environment and Conservation Council and the Agriculture and Resource Management Council of Australia and New Zealand, Canberra, Australia, 2000).
39 Ministry for the Environment. New Zealand Periphyton Guideline: Detecting, Monitoring and Managing Enrichment of Streams. 122 (Ministry for the Environment, Wellington, New Zealand, 2000).
40 Wagenhoff, A., Clapcott, J. E., Lau, K. E. M., Lewis, G. D. & Young, R. G. Identifying congruence in stream assemblage thresholds in response to nutrient and sediment gradients for limit setting. *Ecol. Appl.* **27**, 469-484, doi:doi:10.1002/eap.1457 (2017).
41 Schneider, S. C. & Lindstrøm, E.-A. The periphyton index of trophic status PIT: a new eutrophication metric based on non-diatomaceous benthic algae in Nordic rivers. *Hydrobiologia* **665**, 143-155, doi:10.1007/s10750-011-0614-7 (2011).
42 Oberholster, P. J., Somerset, V. S., Truter, J. C. & Botha, A.-M. The Interplay between Environmental Conditions and Filamentous Algae Mat Formation in Two Agricultural Influenced South African Rivers. *River Res. Appl.* **33**, 388-402, doi:doi:10.1002/rra.3081 (2017).
43 Malo, D. D. & Gelderman, R. H. Portable soil test laboratory results compared to standard soil test values. *Communications in Soil Science & Plant Analysis* **15**, 909-927 (1984).

44 Wolf, A. M. & Baker, D. E. Comparisons of soil test phosphorus by Olsen, Bray I, Mehlich I and Mehlich III methods. *Communications in Soil Science & Plant Analysis* **16**, 467-484 (1985).
45 Batjes, N. H. Overview of soil phosphorus data from a large international soil database. 56 (Wageningen and ISRIC - World Soil Information, Wageningen, The Netherlands, 2011).
46 Toth, G., Jones, A. & Montanarella, L. LUCAS Topsoil Survey methodology, data and results. 141 (Publications Office of the European Union, Luxembourg, 2013).
47 Moody, P. W., Speirs, S. D., Scott, B. J. & Mason, S. D. Soil phosphorus tests I: What soil phosphorus pools and processes do they measure? *Crop and Pasture Science* **64**, 461-468, doi:https://doi.org/10.1071/CP13112 (2013).
48 McKenzie, N. J., Jacquier, D. W., Maschmedt, D. J., Griffin, E. A. & Brough, D. M. The Australian Soil Resource Information System (ASRIS) Technical Specifications. Revised Version 1.6, June 2012. . (CSIRO, Canberra, Australia, 2012).
49 McDowell, R. W. *et al.* Why are median phosphorus concentrations improving in New Zealand streams and rivers? *J. R. Soc. N. Z.* **In press** (2019).
50 Fischer, G. *et al.* Global Agro-ecological Zones Assessment for Agriculture (GAEZ v3.0). 179 (International Institute for Applied Systems Analysis and Food and Agriculture Organization of the United Nations, Laxenburg, Austria and Rome Italy, 2012).
51 Genstat v17.0 (VSNI, Hemel Hempstead, UK, 2015).
52 McDowell, R. W. in *Siol across latitudes* Online at: https://scisoc.confex.com/scisoc/2019sssa/meetingapp.cgi/Paper/115079 (Soil Science Society of America, San Diego CA, 2019).
53 Hengl, T. *et al.* Soil nutrient maps of Sub-Saharan Africa: assessment of soil nutrient content at 250 m spatial resolution using machine learning. *Nutrient Cycling in Agroecosystems* **109**, 77-102, doi:10.1007/s10705-017-9870-x (2017).
